# Does hyperthyroidism worsen prognosis of thyroid carcinoma? A retrospective analysis on 2820 consecutive thyroidectomies

**DOI:** 10.1186/s40463-018-0254-2

**Published:** 2018-01-22

**Authors:** Fabio Medas, Ernico Erdas, Gian Luigi Canu, Alessandro Longheu, Giuseppe Pisano, Massimiliano Tuveri, Pietro Giorgio Calò

**Affiliations:** 1 Department of Surgical Sciences, University of Cagliari, Cittadella Universitaria, SS554, Bivio Sestu, 09042 Monserrato (CA), Italy; 2 Istituto Pancreas, Policlinico Borgo Roma, AOUI Verona, Piazzale L.A. Scuro, 10, 37134 Verona, Italy

**Keywords:** Hyperthyroidism, Thyroid carcinoma, Graves’ disease, Thyroidectomy

## Abstract

**Background:**

Hyperthyroidism is associated with high incidence of thyroid carcinoma; furthermore, tumors arisen in hyperthyroid tissue show an aggressive behavior. Thyroid Stimulating Hormone (TSH) and Thyroid-stimulating antibodies, present in Graves’s disease, seem to play a key role in carcinogenesis and tumoral growth.

**Methods:**

We retrospectively reviewed our series of patients who underwent thyroidectomy for thyroid carcinoma. We compared pathological features and surgical outcomes of hyperthyroid versus euthyroid patients.

**Results:**

From 2007 to 2015, 909 thyroidectomies were performed at our institution for thyroid cancer: 87 patients were hyperthyroid and 822 euthyroid. We observed, in hyperthyroid patients, a higher rate of transient hypoparathyroidism (28.1% vs 13.2%; *p* < 0.01) and of node metastases (12.6% vs 6.1%; *p* = 0.03); also local recurrence rate was higher (5.7% vs 2.5%) even if not statistically significant (*p* = 0.17). Five-year disease free survival rate was significant lower in the same group (89.1% vs 96.6%; *p* = 0.03).

**Conclusion:**

Thyroid cancers in hyperthyroid patients have an aggressive behavior, with high incidence of local invasion and a worse prognosis than euthyroid patients. All hyperthyroid patients should undergo a careful evaluation with ultrasound and scintigraphy; in case of suspicious nodules, an aggressive approach, including thyroidectomy and lymphectomy, is justified. In patients with toxic adenoma, thyroid cancer is uncommon, thus a loboisthmectomy can be safely performed.

**Trial registration number:**

Research registry n. 2670 registered 19 June 2017 (retrospectively registered).

## Background

In the past hyperthyroidism was considered a protective factor for thyroid carcinoma [1]. Nevertheless, since the Fifties some studies [2, 3] reported a high incidence of thyroid carcinoma in patients affected from Graves’ disease. From the ‘90s several authors [4–7] have suggested that not only thyroid carcinoma is frequently associated with hyperthyroidism, especially with Graves’ disease, but it also has an aggressive behavior. In this scenario, TSH seems to play a key role: it is known as the most important factor stimulating normal thyroid tissue growth, but it has been reported that it can also stimulate neoplastic thyroid tissue, that contains functional TSH receptors. Filetti and Mazzaferri [4, 8] have observed that thyroid-stimulating antibodies, that are similar to TSH and are present in Graves’ disease, may promote tumor growth by activating TSH receptors.

The aim of this retrospective study is to evaluate pathological features and clinical behavior of thyroid carcinoma arising in hyperthyroid patients, and to assess whether there are relevant differences compared with euthyroid patients.

## Methods

### Study design

After ethical approval by local ethics committee (Indipendent Ethic Committee, University of Cagliari), we conducted a retrospective study on patients who had undergone thyroidectomy in our Department of General and Endocrine Surgery (University of Cagliari) between January 2007 and December 2015 with pathological diagnosis of thyroid carcinoma. Based on metabolic status, patients were divided into two groups: hyperthyroid patients, including Graves’s disease, Toxic Adenoma and Toxic Multinodular Goiter were included in Group A, while euthyroid patients were included in Group B.

All the patients included in group A had received diagnosis of hyperthyroidism, defined as low serum TSH level (< 0.4 mIU/L) with high or normal (subclinic hyperthyroidism) free T4 and free T3 serum levels.

Patients demographics, preoperative data, pathological findings, postoperative complications (including recurrent nerve injury and hypoparathyroidism) and locoregional or distant recurrence were recorded.

### Preoperative assessment

All the patients included in the study underwent preoperative physical examination, dosage of serum thyroid hormones including TSH, Tg and anti-Tg antibody and high-resolution ultrasonography of the neck. At the time of surgery, all patients had normal FT3 and FT4 serum levels, including those in group A due to assumption of antithyroid drugs.

In case of suspicious nodules, fine needle aspiration cytology (FNAC) was performed. Preoperative fibrolaryngoscopy was routinely performed to assess vocal folds mobility. In hyperthyroid patients a thyroid scintigraphy was routinely performed.

Surgery. Surgical procedure consisted in extracapsular total thyroidectomy; recurrent laryngeal nerves were routinely exposed until their insertion in larynx, and any attempt to preserve parathyroid glands was performed. In patients with preoperative or intraoperative suspicion of lymph node metastases, central node dissection or radical lateral neck dissection was performed to achieve curative treatment. A subfascial drainage was routinely used.

### Postoperative management

Serum calcium level was assayed on first and second postoperative day to promptly detect hypocalcemia: in this case, diagnosis of hypoparathyroidism was confirmed in case of PTH < 10 pg/ml (normal range = 10-65 pg/ml). Drainage was usually removed in second postoperative day. In case of suspected recurrent nerve injury, a fibrolaryngoscopy was performed to assess vocal cord mobility.

### Pathological examination

Thyroid cancer was classified according to TNM classification of AJCC (7th edition, 2010). Locally invasive carcinoma was defined as T3 or T4 based on TNM staging.

### Follow up

Suppressive L-Thyroxine therapy was routinely administrated. Serum Tg and Tg-antibody levels were assayed every 6 months together with neck ultrasound (US). Diagnosis of recurrent disease was confirmed with US-guided FNC of lymph nodes and Tg washing of FNC aspirates. Patients considered disease-free underwent Tg detection after rhTSH stimulation and neck US.

Hypoparathyroidism and nerve palsy were defined permanent if persisting for more than 12 months after surgery.

### Statistical analysis

Chi-squared test was used for categorical data and T-Test for continuous variables. Results were considered statistically significant if *p* value was ≤0.05. Continuous data are reported as the mean value ± standard error of the mean. Calculations were performed with MedCalc ® 12.7.0.0.

## Results

Between January 2007 and December 2015, 2820 patients underwent thyroidectomy at our department: 423 were hyperthyroid and 2398 euthyroid. The incidence of thyroid carcinoma was 20.6% in hyperthyroid patients (*n* = 87) and 34.3% in euthyroid (*n* = 822). In total, 909 patients with diagnosis of thyroid carcinoma were included in the present study. The patients were divided into two groups in accordance with criteria mentioned above: 87 patients were included in group A and 822 in group B.

### Demographic data and surgical treatment (Table 1)

Group A: Preoperative diagnosis was Graves’ disease in 45 (51.7%) patients, Toxic Multinodular Goiter in 37 (42.5%) and Toxic Adenoma in 5 (5.7%). There were 23 (26.4%) males and 64 (73.6%) females with a mean age of 51.5 ± 14.2 years. Surgical procedure consisted in total thyroidectomy (TT) in 72 (82.8%) cases, associated to central lymph node dissection (CLND) in 7 (8%) patients and lateral neck dissection in 3 (3.4%); a loboisthmectomy was performed in 5 (5.7%) cases. Mean operative time was 107.7 ± 33.4 min.

**Table 1:** Demographic data and surgical procedure

	Group A (*n* = 87)	Group B (n = 822)	p value
Male	23 (26.4%)	161 (19.6%)	0.17
Female	64 (73.6%)	661 (80.4%)	
Age (years)	51.5 ± 14.2	51.4 ± 15.4	0.81
Surgical procedure			
TT	72 (82.8%)	669 (81.4%)	0.67
TT + CLND	7 (8.0%)	96 (11.7%)	
TT + LND	3 (3.4%)	23 (2.8%)	
Loboisthmectomy	5 (5.7%)	34 (4.1%)	
Operative time (minutes)	107.7 ± 33.4	99.3 ± 28.8	0.61

*TT* Total thyroidectomy, *CLND* central lymph node dissection, *LND* lateral neck dissection

Group B: There were 161 (19.6%) males and 661 (80.4%) females with a men age of 51.5 ± 15.4 years. The patients underwent to total thyroidectomy (TT) in 669 (81.4%) cases, associated to CLND in 96 (11.7%) patients and lateral neck dissection in 23 (2.8%). A loboisthmectomy was performed in 34 (4.1%) cases. Mean operative time was 99.3 ± 28.8 min.

### Surgical outcomes and follow up (Table 2)

Group A. Mean postoperative stay was 2.2 ± 0.8 days; transient hypoparathyroidism was observed in 29 (28.1%) patients and persistent in 4 (4.5%); recurrent nerve injury occurred in 2 (2.2%) cases. Mean follow up was 59.3 ± 29.2 months. Local recurrence was observed in 5 (5.7%) patients with a 5-year disease free survival rate of 89.1%.

**Table 2:** Surgical outcomes and follow up

	Group A (n = 87)	Group B (n = 822)	*p* value
Postoperative stay (days)	2.2 ± 0.8	2.3 ± 1.2	0.81
Follow-up (months)	59.3 ± 29.24	62.1 ± 19.8	0.77
Transient hypoparathyroidism	29 (28.1%)	109 (13.2%)	<0.01
Permanent hypoparathyroidism	4 (4.5%)	31 (3.7%)	0.93
Recurrent nerve injury	2 (2.2%)	14 (1.7%)	0.97
Local recurrence	5 (5.7%)	21 (2.5%)	0.17
5-year disease free survival rate	89.1%^a^	96.6%^a^	0.03

^a^ On 46 patients (Group A) and 621 patients (group B) with at least 5 years of followup

Group B: Mean postoperative stay was 2.3 ± 1.2 days. Transient hypoparathyroidism was reported in 109 (13.2%) patients and permanent in 31 (3.7%). Transient recurrent nerve injury was observed in 14 (1.7%) cases. Local recurrence occurred in 21 (2.5%) patients with a 5-year disease free survival rate of 96.6%.

### Pathologic data (Table 3)

Group A. Mean tumor size was 2.31 ± 0.9 cm. Papillary thyroid carcinoma was reported in 59 (67.8%) patients, follicular variant of papillary carcinoma in 17 (19.5%), tall cell carcinoma in 3 (3.4%), follicular carcinoma in 5 (5.7%) and Hurtle cell carcinoma in 3 (3.4%). A locally invasive carcinoma was found in 15 (17.2%) cases and a multicentric neoplasia in 25 (28.7%). Node metastases were observed in 11 (12.6%) patients.

**Table 3:** Pathologic data

	Group A (n = 87)	Group B (n = 822)	*p* value
Tumor size (mm)	2.31 ± 0.9	2.35 ± 1.15	0.74
Locally invasive carcinoma	15 (17.2%)	194 (23.6%)	0.22
Multicentric carcinoma	25 (28.7%)	200 (24.3%)	0.43
Node metastasis	11 (12.6%)	50 (6.1%)	0.03
Histopathologic diagnosis			
PTC	59 (67.8%)	464 (56.4%)	0.19
Follicular variant of PTC	17 (19.5%)	190 (23.1%)	
Tall cell carcinoma	3 (3.4%)	23 (2.8%)	
Follicular carcinoma	5 (5.7%)	110 (13.4%)	
Hurtle cell carcinoma	3 (3.4%)	35 (4.3%)	

*PTC* Papillary thyroid carcinoma

Group B. Mean tumor size was 2.35 ± 1.15 cm. Papillary thyroid carcinoma was reported in 464 (56.4%) patients, follicular variant of papillary carcinoma in 190 (23.1%), tall cell carcinoma in 23 (2.8%), follicular carcinoma in 110 (13.4%) and Hurtle cell carcinoma in 35 (4.3%). A locally invasive carcinoma was reported in 194 (23.6%) patients and a multicentric tumor in 200 (24.3%). Node metastases were observed in 50 (6.1%) patients.

### Comparison between the groups

Demographic data, surgical procedure and operative time were similar between the groups. Incidence of transient postoperative hypoparathyroidism was higher in Group A (*p* < 0.01), whereas permanent was similar. Pathologic features were similar between the groups, except the incidence of node metastases that was higher in Group A (12.6% vs 6.1%; *p* = 0.03). Local recurrence was more than twice in group A as much in group B, but not statistically significant (5.7% vs 2.5%; p 0.17). Five-year disease free survival rate was significant lower in Group A (89.1% vs 96.6%; *p* = 0.03).

## Discussion

Before 1950 few cases of association between hyperthyroidism and carcinoma were reported, so that some authors suggested that hyperthyroidism could be a cancer-protective factor [1, 9]. Nevertheless, in the last decades several studies [4–7] have reported that not only thyroid carcinoma occurs with high frequency in hyperthyroid patients, but it also has a more aggressive behavior than usual. Filetti [8] noted that TSH stimulates growth of both normal and cancer cells, and that there are similarities between TSH and thyroid-stimulating antibodies in Graves’ disease. In fact both activate adenylate cyclase and phospholipase C cascade, with mitogenic and antiapoptotic effects, causing normal thyroid tissue to become hyperplastic and hyperfunctional; furthermore, thyroid cancer cells contain functional TSH receptors. These evidences support the hypothesis that thyroid-stimulating antibodies and TSH play a key role in carcinoma’s pathogenesis.

The prevalence of thyroid carcinoma in hyperthyroid patients varies widely in literature, ranging from 0.5 to over 20% [2, 3, 9–22]. The reason of this wide difference is probably related to patients’ selection for surgery, type of surgery and geographical variation of incidence of thyroid carcinoma; in addition, the incidence is higher in retrospective studies that only include patients who underwent thyroidectomy, and lower in studies that include also patients who did not underwent surgery.

Anyway, most of the authors report a higher incidence of thyroid carcinoma in hyperthyroid than euthyroid patients. Sokal [2] found a 20-fold increase of thyroid carcinoma incidence in patients with hyperthyroidism. Yeh [23], in a large population-based cohort study, analyzed data of 1 million of patients from Taiwan’s National Health Insurance database and reported an increased risk of head and neck cancer in patients with hyperthyroidism, especially for thyroid carcinoma.

We observed in our study a high incidence of carcinoma in hyperthyroid patients (20.6%) but lower than euthyroid patients (34.3%). About this finding, it’s important to note that ours is a iodine-deficiency region, thus subclinic hypothyroidism, resulting in chronic increased serum TSH levels, may explain the high incidence of thyroid carcinoma in euthyroid patients.

Many authors [7, 9, 14] have reported a high frequency of aggressive subtypes of thyroid carcinoma in hyperthyroidism, being larger, more often multicentric, locally invasive or metastatic.

Belfiore [14, 15] described a higher incidence of thyroid carcinoma in Grave’s disease than in toxic adenoma. In addition, he found that tumor with aggressive behavior were more frequent in Graves’ disease, of intermediate frequency in euthyroid patients and uncommon in toxic adenoma patients. These findings support the hypothesis that TSH and thyroid-stimulating antibodies play a key role in tumor genesis and in promoting growth and metastatic spread of thyroid cancer. In fact tumors with an aggressive behavior are less frequent in patients with low or suppressed sereum TSH levels, and, by the other side, more frequent in those with chronic stimulation of TSH receptor by thyroid stimulating antibodies.

These observations are consistent with our findings: we observed an aggressive behavior of tumors arisen in hyperthyroid patients, with a higher incidence of node metastases than in euthyroid patients (12.6% vs 6.1%) and with a worse prognosis (5-year disease free survival of 89.1% compared with 96.6% of euthyroid patients). We did not find larger tumors in hyperthyroid patients, but this could be related, as stated earlier, to chronic TSH stimulation in euthyroid patients due to iodine deficiency.

An Italian study [24] with a long follow-up found not only a lower disease free survival but also a higher disease-specific mortality in patients with thyroid cancer and Grave’s disease than in euthyroid patients.

Furthermore, it has been reported that patients with Grave’s disease and a palpable nodule are at high risk to have a thyroid carcinoma: in these patients the incidence of thyroid carcinoma reaches 50% [12, 14, 15, 17, 25, 26]. For these reasons, a palpable nodule found in the course of Grave’s disease should be strongly considered for surgery.

A fine needle aspiration cytology (FNAC) from nodules could be taken into account, but diagnosis is often difficult due to cytomorphologic changes as a consequence of the disease and of antithyroid drug treatment. In addition, radioiodine treatment produce cellular atypia that can lead to erroneous diagnosis of malignancy; therefore FNAC should be performed prior to radioiodine therapy.

The extent of surgery in hyperthyroid patients with preoperative diagnosis of carcinoma has been matter of debate. Because of the aggressive behavior of the tumor, an aggressive approach, including total thyroidectomy with prophylactic central lymph node dissection, seems justified in these cases, following current trend in thyroid surgery [27–33]. By the other side, patients with Grave’s disease or multinodular toxic goiter in whom no suspicion of carcinoma exists, should be treated with only total thyroidectomy.

Patients with toxic adenoma are usually treated with loboisthmectomy. If pathological examination demonstrates a carcinoma, removal of contralateral lobe is not necessary in case of small (<1 cm), unifocal, low-risk, intrathyroidal nodules and no lymph node metastases. In our series, we found only 5 cases of carcinoma arisen in patients with toxic adenoma; all of them had undergone loboisthmectomy and diagnosis of carcinoma was incidental. Pathologic diagnosis was in all the cases papillary microcarcinoma, thus none of them required a subsequent contralateral lobectomy.

According to literature [34–37], we observed in our series a higher incidence of transient hypoparathyroidism in hyperthyroid patients (28.1%) than in euthyroid (13.2%; *p* < 0.01). Common causes of postoperative hypocalcemia are damage, devascularization or inadvertent removal of parathyroid glands, but another cause that has been postulated for hyperthyroid patients is “hungry bone syndrome”, due to postoperative reversal of thyrotoxic osteodystrophy [38]. It’s therefore necessary a careful follow-up with serum calcium measurement to promptly detect hypocalcemia, and, where appropriate, adequate calcium supplementation should be administrated to prevent hypocalcemia-related symptoms.

Finally, it should be pointed out that, being aware of the aggressive behavior of thyroid cancer in hyperthyroid patients, a more aggressive surgical approach could have been carried out in hyperthyroid rather than euthyroid patients, resulting, for example, in more extensive lymphectomies with higher incidence of complications and even of detected node metastases; thus, this could represent a bias of this study.

## Conclusion

Thyroid cancers arisen in hyperthyroid gland have an aggressive behavior, with high incidence of local invasion and a worse prognosis than euthyroid patients, especially in patients with Graves’ disease. A careful evaluation with ultrasound and scintigraphy should be routinely performed in hyperthyroid patients; in case of suspicious nodules, an aggressive approach, including thyroidectomy and lymphectomy, is justified. A FNAC of the nodule could be helpful, even if its results are often inconclusive. Thyroid cancer is uncommon in patients with toxic adenoma, thus these patients can be safely treated with a loboisthmectomy.

## Abbreviations

FNAC: Fine Needle Aspiration Cytology
Tg: Thyroglobulin
TSH: Thyroid Stimulating Hormone
US: Ultrasound
